# Decomposing Effects of Time on Task Reveals an Anteroposterior Gradient of Perceptual Decision Regions

**DOI:** 10.1371/journal.pone.0072074

**Published:** 2013-08-19

**Authors:** Bradley R. Buchsbaum, Drew T. Erickson, Andrew S. Kayser

**Affiliations:** 1 Department of Psychology, University of Toronto, Toronto, Ontario, Canada; 2 Department of Neurology, The University of California at San Francisco, San Francisco, California, United States of America; 3 Department of Neurology, Veterans Affairs Northern California Health Care System, Martinez, California, United States of America; University of California, Davis, United States of America

## Abstract

In perceptual decision making, the selection of an appropriate action depends critically on an organism’s ability to use sensory inputs to accumulate evidence for a decision. However, differentiating decision-related processes from effects of “time on task” can be difficult. Here we combine the response signal paradigm, in which the experimenter rather than the subject dictates the time of the response, and independent components analysis (ICA) to search for signatures consistent with time on task and decision making, respectively, throughout the brain. Using this novel approach, we identify two such independent components from BOLD activity related to a random dot motion task: one sensitive to the main effect of stimulus duration, and one to both the main effect of motion coherence and its interaction with duration. Furthermore, we demonstrate that these two components are expressed differently throughout the brain, with activity in occipital regions most reflective of the former, activity within intraparietal sulcus modulated by both factors, and more anterior regions including the anterior insula, pre-SMA, and inferior frontal sulcus driven almost exclusively by the latter. Consistent with these ICA findings, cluster analysis identifies a posterior-to-anterior gradient that differentiates regions sensitive to time on task from regions whose activity is strongly tied to motion coherence. Together, these findings demonstrate that progressively more anterior regions are likely to participate in progressively more proximate decision-related processes.

## Introduction

Perceptual decision-making is a fundamental cognitive process in which sensory input guides the selection of one of many possible actions. This translation from sensation to action is thought to occur by a mechanism in which sensory evidence accumulates over time until the threshold for a decision is reached. Importantly, such a process has been observed in neurons whose firing rates increase proportionally with the strength of the sensory stimulus in regions including the lateral intraparietal cortex (LIP) [1], the frontal eye fields [2], the caudate [3], and the premotor cortex [4]. Thus, these studies suggest that many regions are involved in evidence accumulation and decision-making networks.

Further defining these networks is a problem well suited to the whole-brain coverage provided by functional MRI studies. Human work has identified a number of brain regions whose activity varies with the amount of evidence available for perceptual decisions, including the middle intraparietal sulcus (the homologue of macaque LIP [5]), midline motor areas, dorsolateral frontal regions, and the anterior insula [6], [7], [8], [9], [10]. However, such studies have not always been consistent in their identification of the key areas for evidence accumulation, nor have the identified brain networks in human research always aligned with those identified in electrophysiological studies with macaques (e.g. with respect to the participation of lateral frontal areas in evidence accumulation).

Why do these studies diverge? Potentially problematic for human studies are the correlated contributions of decision-related processes including evidence accumulation and what has often been referred to as “time on task” – i.e. the idea that a certain amount of non-specific brain activity can be attributed merely to the passage of time, or a “duty-cycle.” This issue arises because of the correlation between stimulus strength, the decision process, and reaction time: as the strength of the stimulus increases, evidence accumulation occurs more quickly, and reaction time decreases. For a dependent measure such as regional brain activity, the question of whether activity is correlated with a decision process such as evidence accumulation, or simply to the duration of the trial, becomes confounded. A sensory region, for example, might show activity that scales with motion coherence, but that is actually related solely to the duration of bottom-up attention captured by the stimulus on the screen. On the other hand, a region directly involved in the decision process may also show independent effects of time on task that, if not distinguished, might obscure its participation in the decision.

Previous work has attempted to address the influence of time on task by exploiting variability in subjects’ reaction times. A study by Yarkoni and colleagues [11], for example, collected data from five different cognitive tasks and searched for brain regions whose activity correlated with subject reaction times across task. Similarly, Grinband and colleagues [12], as well as Weissman and Carp [13], investigated a more focused question: whether activity in the medial frontal cortex correlated more strongly with the presence of response conflict or reaction time/time on task. Broadly speaking, the above studies were concerned with distinguishing activity related to the duration of cognitive processing from activity related to specific cognitive processes. In contrast with our approach here, however, all of these studies focused on reaction times, rather than stimulus duration, and were thus unable to distinguish the relative contribution of stimulus-bound non-decision processes.

In non-human primates, regions implicated in visual perceptual decision making have also been evaluated. Yang and Lisberger [14] showed, for example, that responses of neurons in the motion-sensitive region MT to dot motion stimuli adapt, but remain robust, over stimulus durations of eight seconds. In contrast, direct studies of the effects of stimulus duration in other brain areas are less common. In a delayed eye movement task, a subject of neurons in the lateral intraparietal area (LIP) of the macaque demonstrated activity that signaled both elapsed time during the delay and the probability that a movement signal would arrive [15]. In areas 8 and 46 of the lateral frontal cortex, approximately 20% of neurons encoded the duration of at least one of two temporal stimuli in a two-sample duration discrimination task [16]. While these studies indicate that duration-sensitive neural responses can be found in temporal, parietal, and frontal cortices, they did not directly address the issue of stimulus duration as an independent factor in a separate perceptual decision.

This thorny issue of time on task has led to several alternative approaches to identifying decision-related regions in perceptual decision making tasks in humans. Different studies have identified potential decision-related processes by searching for a parametric effect across levels of stimulus discriminability [10], early-trial differences in the BOLD response between high and low coherence stimuli [7], sensitivity to both sensory and response factors such as errors [8], and changes in time course during extended recognition paradigms [9], among others. None of the above approaches, however, has explicitly varied stimulus duration/response timing and stimulus strength to assess the independent influence of time on task that cannot be attributed to task difficulty per se. This manipulation is especially important because while a decision-related region should be sensitive to both the amount of evidence and the duration over which evidence is accrued [17], it should not be linked solely to the duration of stimulus presentation. In other words, when stimulus duration effects do occur in a decision-related region, in order to influence the decision they should be expressed through an interaction with stimulus strength. For example, a progressively greater duration of stimulus presentation should differentially affect accuracy and neural activity in response to intermediate motion coherence values (e.g. 10%) but not extreme values (e.g. 0% and 100%), leading to a behavioral and neural interaction. More specifically, as predicted by the diffusion model [18] progressively longer stimulus durations differentially affect the ability of evidence-accumulating neurons to reach threshold in response to low rather than high motion coherence values. In contrast, a region whose activity does not depend on motion coherence, but does depend on stimulus duration, could not provide the basis for decisions based on sensory evidence.

To address these hypotheses, here we pursue a novel approach combining a response signal paradigm [19], [20] that explicitly controls time on task factors with a methodological approach, independent components analysis, that enables us to distinguish networks and regions sensitive to duration, motion coherence, and their interaction. In our paradigm, response time and motion coherence are independently manipulated to identify brain regions whose activity covaries with the available evidence. Thus, rather than allowing the subject to freely decide when to make a response on each trial according to his or her own decision criteria, the experimenter controls the timing of the response in a fashion that varies independently of task difficulty. On each trial, the subject awaits a “response signal”, variably timed across trials, indicating when a response should be made irrespective of the state of evidence accumulation. By ensuring that subjects cannot easily predict when the response signal will occur [15] – thus encouraging uniform attention across each trial – this design investigates the hypothesis that regions involved in decision-related processes such as evidence accumulation should be sensitive to both motion coherence and *decision* duration, but that regions involved in sensory processing should be most sensitive to *stimulus* duration. Moreover, following ideas derived from human lesion studies [21], [22] that anterior regions are more likely to be engaged in decision-related processes, while posterior regions are more likely to be engaged in stimulus perception, we directly investigate the prediction that sensitivity to both factors defines an anterior-posterior gradient across the brain.

## Materials and Methods

### Ethics Statement

This study was approved by the Committee for the Protection of Human Subjects (CPHS) at the University of California, Berkeley. Five subjects (ages 22–38; 3 male) participated in the study and gave written informed consent in accordance with the Declaration of Helsinki and CPHS approval. All subjects had normal neural anatomy as assessed by a neurologist (A.S.K.), were right-handed, and had normal or corrected-to-normal vision.

### Task Design

Before each scanning session, subjects were trained on the task for a minimum of six 1-hour practice runs to reduce both the number of invalid trials (see below) and learning effects in the scanner. Once trained, all subjects underwent a minimum of six 1-hour fMRI sessions, each of which consisted of six runs of 72 trials for a total of 6×6×72 = 2592 trials. Three subjects participated in an additional three 1-hour fMRI sessions, resulting in a total of 9×6×72 = 3888 trials for these three subjects.

Subjects performed a visual dot-motion task in which they viewed a variable proportion of coherent dot motion on a background of randomly moving dots. They were required to identify the direction of motion (either leftward or rightward) as quickly and accurately as possible under a time constraint that varied on a trial-by-trial basis. At the beginning of each trial, a bright central fixation cross was dimmed to indicate the onset of the dot-motion stimulus. Subjects viewed the stimulus until the response signal occurred (in this case, stimulus offset), at which point the fixation cross brightened and turned green. Subjects were required to make a button-press response within 350 milliseconds after the onset of the response signal. In order to prevent subjects from predicting the timing of the response signal, we selected a response signal probability function that equated the hazard rate *h(t)* across durations *t*:
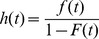



where *f(t)* is the probability that the response signal occurs at time *t*, and *F(t)* is the cumulative distribution 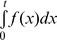 from trial onset to time *t* [15]. This function ensures that the ratio of the probability that the trial ends at time *t*, *f(t)*, to the probability that the trial ends later, 1– *F(t)*, is approximately constant across time. For this purpose, we chose a gamma distribution that produced a median stimulus duration of 0.81 seconds, with a range from 0.22 seconds to 3.3 seconds. Similarly, a gamma distribution defined the motion coherence stimulus, which ranged from 0% to 100% with a median of 13.1%. Both dot-motion coherence and the direction of motion (either leftward or rightward) were consistent within a trial and varied independently across trials. At the end of the 350ms response interval, the central fixation cross reverted from green to white and an interstimulus interval varying between 4000–12000 ms began. Maintenance of fixation was ensured as in our previous studies during sessions outside the scanner [8], [23]. Three subjects were trained in one or both of these previous studies; two subjects were trained to the same eye movement criteria for the current task during sessions prior to MRI scanning. Eye movement data were not obtained during scanner sessions themselves.

For fMRI sessions, the ordering of dot-motion trials was computed using OptSeq (http://surfer.nmr.mgh.harvard.edu/optseq/) [24]. Stimuli were programmed in Matlab in the PsychToolbox environment [25], [26], adapted from code originally written by McKinley & Shadlen and downloaded from the PsychToolbox website (http://psychtoolbox.org/PTB-2/). All characteristics of the dot motion display were unchanged from our previous studies [8], [23], with the exception that the duration of the stimulus was variable as described above.

### MRI Scanning

MRI scanning was conducted on a Siemens MAGNETOM Trio 3T MR Scanner at the Henry H. Wheeler, Jr. Brain Imaging Center at the University of California, Berkeley. Anatomical images consisted of 160 slices acquired using a T1-weighted MP-RAGE protocol (TR = 2300 ms, TE = 2.98 ms, FOV = 256 mm, matrix size = 256×256, voxel size 1×1×1 mm). Functional images consisted of 24 slices acquired with a gradient echoplanar imaging protocol (TR = 1370 ms, TE = 27 ms, FOV = 225 mm, matrix size = 96×96, voxel size 2.3×2.3×3.5 mm). A projector (Avotec SV-6011, http://www.avotec.org/) was used to display the image on a translucent screen placed within the scanner bore behind the head coil. A mirror was used to allow the subject to see the display. The distance from the subject’s eye to the screen was 28 cm.

### fMRI Preprocessing

fMRI functional images were converted to 4D NIfTI format and corrected for slice-timing offsets using SPM5 (http://www.fil.ion.ucl.ac.uk). Motion correction was carried out using the AFNI program *3dvolreg* with a reference volume specified as the mean image of the first run in the series. Images were then smoothed with a 6mm FWHM Gaussian kernel. Co-registration was performed with the AFNI program *3dAllineate* using the local Pearson correlation cost function optimized for fMRI to structural MRI alignment. The inverse transformation was then used to warp the high resolution MRI to the functional image space, after which it served as an anatomical underlay for the display of statistical parametric maps.

### fMRI Data Analysis

Voxel-wise fMRI statistical analyses were carried out for each subject using the general linear model framework implemented in the AFNI program *3dDeconvolve*. To assess the overall effect of motion coherence and duration, each of these factors was divided into 10 bins containing equal numbers of trials. In total, 10×10 = 100 separate condition-specific regressors were derived by convolving a gamma probability density function (peak = 6 s) with a vector of stimulus onsets for each of the conditions. As expected based on the total number of trials per subject, each bin contained 25 or more trials. Mean values for the 10 coherence bins were 1.3, 3.9, 6.3, 8.6, 11.4, 15.2, 22.9, 40.4, 64.2, and 88.0 (in percent coherence); mean values for the 10 duration bins were 0.26, 0.34, 0.46, 0.57, 0.72, 0.88, 1.11, 1.41, 1.90, and 2.77 (in seconds).

The peak height for the convolved hemodynamic function was constrained to remain constant across all task durations tested. This approach ensured that increases in stimulus duration were captured by the beta value, rather than within the regressor itself. In brief, the convolution of a constant HRF with increasing stimulus duration leads to regressors with progressively increasing amplitudes. As a result, the effect of stimulus duration on the beta values would be captured within the regressor time course, not by the beta value. Therefore, in regions sensitive only to duration, beta values might remain constant as stimulus duration increases. To avoid spurious null results (i.e. ones in which a constant beta was misinterpreted as a stimulus duration effect), we constrained peak height to ensure that betas sensitive to duration would demonstrate a parametric effect of duration in regions sensitive to stimulus duration.

Our analyses included both correct and error trials. Although error trials can occur for multiple reasons – e.g. transient failures of sensory input due to eye closure or inattention, or failures of motor output due to incorrect mappings from stimulus to response – they are nonetheless tied to the decision and not solely to the duration of the stimulus. Therefore, they also potentially differentiate an independent component related to the decision process from one related to time on task, leading to a stronger test of our hypotheses. Furthermore, any heterogeneity in error responses would likely only add noise to our analyses, and removing them would add imbalance to the number of trials in each condition. It should also be noted that subjects did not receive performance feedback, so that subjects were rarely if ever aware of the commission of an error, especially during the difficult trials (less than 5% motion coherence) wherein the bulk of errors occurred. Therefore, error trials were incorporated into the independent components analysis.

Tests of linear trends were carried out using the contrast vectors applied to the estimated beta coefficients in each voxel for each motion coherence × duration bin. The resulting t-statistics were spatially normalized to MNI space. The estimated beta coefficients for each bin were then subjected to a group ICA analysis (see next section). To avoid a selection bias in the definition of ROIs, we used the AFNI program *3dmaxima* to generate functional ROIs from a group analysis of the main effect of task (i.e. group activity collapsed across coherence and duration manipulations), thresholded at a T statistic of 5. Regions consisted of spheres of radius 9 mm whose boundaries were separated from each other by at least 3mm. ROI labels were defined as in our previous work [8]. In particular, the IPS was subdivided into anterior, medial and posterior subdivisions following the criteria of Stark and Zohary [27]. The anterior IPS was defined as the anterior-most third of the sulcus, the medial IPS was defined as the dorsal-most half of the posterior portion of the IPS, and the posterior IPS was defined as the ventral-most half of the posterior portion of the IPS.

Time courses for ROIs were estimated via the AFNI program *3dDeconvolve* using a set of 8 b-spline basis functions spanning the interval from 0 to 18 seconds post-stimulus. Because of the large number of calculations required for time courses, the motion coherence and duration variables were both divided into four quartiles rather than 10 deciles, yielding 16 bins for which time courses were estimated. The time courses were then averaged within each of the ROIs and submitted to a two-way repeated measures ANOVA to test for effects of motion, duration, and the motion × duration interaction.

### Independent Components Analysis

To search for patterns of activity consistent with evidence accumulation, we applied independent components analysis (ICA) to the whole brain as implemented in the *Melodic* program distributed with FSL. In brief, ICA attempts to separate the additive, statistically independent, non-Gaussian sources that together comprise the data of interest – in this case, not the raw time series, but the beta values generated by our GLM analysis. Unlike a voxel-wise ANOVA, this multivariate approach permits us to identify networks of brain regions whose activity correlates with each of the independent components derived from the data. Thus, the 100 beta volumes (comprising the 10×10 factorial combination of duration and motion coherence conditions) produced for each of our 5 subjects were entered into a multi-subject ICA analysis, from which independent components representing strongly duration-dependent and strongly motion-coherent dependent responses were identified. Specifically, the components that demonstrated the most significant parametric modulation by (1) duration and (2) motion coherence were selected for further analysis. (Of note, as indicated in Results, component 2 showed both the strongest effect of motion coherence and the strongest interaction between motion coherence and duration.) For each of these selected components, the statistical significance of the whole-brain spatial map was determined using mixture modeling and an alternative hypothesis testing approach as implemented in *Melodic* [28]. As noted previously, to evaluate how different brain regions reflected each of these independent components, we applied the AFNI program *3dMaxima* to the main effect of task, resulting in the generation of 41 regions of interest. For each of these ROIs, the 10 voxels demonstrating the largest values for each component were averaged together to produce a summary value. This approach was used in order to avoid including voxels that were not spanned by the spatial maps corresponding to each component, and thereby to provide maximal sensitivity for the relative contributions of each component (see Table 1).

**Table 1:** Regions of interest, as indicated by names, MNI coordinates, Z-scores for the independent duration and motion coherence components, and cluster membership.

ROI	Side	MNI(x)	MNI(y)	MNI(z)	Duration	Motion	Cluster
SMA	L	−4	1	61	7.52	4.09	Green
Occ	R	15	−90	−9	18.82	0.00	Navy
IFS	R	54	7	40	5.25	5.97	Cyan
Occ	L	−7	−96	−9	16.46	0.00	Navy
FEF	L	−31	−6	55	4.07	8.10	Cyan
FEF	R	36	−6	52	5.03	8.06	Cyan
MT+	L	−46	−75	7	9.11	4.03	Green
mIPS	L	−25	−60	55	7.16	6.48	Cyan
pIPS	L	−25	−81	−25	6.91	3.87	Green
IFS	L	−46	4	31	0.71	6.93	Cyan
SOG	R	24	−96	19	12.21	0.00	Navy
MT+	R	48	−72	4	9.31	3.08	Green
pSMA	L	−7	19	49	0.24	8.29	Cyan
IPL	L	−49	−42	58	0.25	4.20	Green
Fus	R	27	−72	−6	6.96	0.00	Orange
mIPS	R	33	−57	55	3.96	4.76	Green
pIPS	R	30	−75	28	5.13	3.48	Green
Crb	R	24	−54	21	1.97	0.00	Orange
aINS	R	36	22	10	0.00	6.23	Cyan
Fus	L	−25	−75	−12	7.56	0.00	Orange
PoG	L	−64	−21	28	2.53	0.24	Orange
aINS	L	−31	25	7	0.00	7.93	Cyan
Thal	L	−10	−18	10	2.97	0.00	Orange
Put	L	−25	−3	7	5.99	0.00	Orange
sPT	R	54	−42	16	2.37	3.27	Green
Put	R	24	4	7	5.69	0.00	Orange
aIPS	R	45	−39	49	1.00	5.54	Cyan
sPT	L	−46	−54	19	0.00	2.64	Orange
PoG	R	66	−15	22	0.23	0.99	Orange
dPM	L	−16	−18	70	3.52	0.22	Orange
aOcc	L	−43	−60	−15	0.00	0.00	Orange
IFG	R	51	7	4	3.53	0.92	Orange
dPM	R	21	1	70	0.00	3.15	Orange
aSFG	R	36	58	28	0.00	0.00	Orange
aSFG	L	−34	55	31	0.00	0.00	Orange
PCC	R	3	−33	28	0.00	0.45	Orange
Cun	R	9	−72	55	0.00	3.02	Orange
Crb	R	6	−51	−6	0.00	0.00	Orange
SPL	L	−34	−48	73	0.00	0.00	Orange
Cun	R	9	−48	55	0.00	0.00	Orange
aPFC	L	−34	49	4	0.00	1.31	Orange

Abbreviations: aINS = anterior insula, aIPS = anterior intraparietal sulcus, aOcc = anterior occiptal region, aPFC = anterior prefrontal cortex, Crb = cerebellum, Cun = cuneus, dPM = dorsal premotor cortex, FEF = frontal eye fields, Fus = fusiform gyrus, IFG = inferior frontal gyrus, IFS = inferior frontal sulcus, IPL = inferior parietal lobule, mIPS = middle intraparietal sulcus, MT+ = middle temporal region, Occ = occipital pole, PCC = posterior cingulate cortex, pIPS = posterior intraparietal sulcus, PoG = post-central gyrus, Put = putamen, SMA = supplementary motor area, SOG = superior occipital gyrus, SPL = superior parietal lobule, sPT = superior planum temporale, STG = superior temporal gyrus, Thal = thalamus.

### Identification of Functionally Related Clusters

To identify regions performing potentially related functions, two analyses were performed. First, the 2-dimensional space defined by the duration and coherence-sensitive motion ICA components was projected onto vectors spanning 360 degrees within this space. To evaluate whether the ordering defined by this projection corresponded to a neuroanatomical (specifically, anterior-posterior) organization of these same areas, we subjected the projection to a non-parametric correlation (Kendall’s tau) with the ordering defined by the Y-coordinate for the centroid of each ROI within MNI space. The strongest correlation was assessed for both direction and statistical significance.

To quantify the relatedness of different regions within the 2-dimensional space defined by the independent components, we applied K-means clustering. In this method, the observations are divided into *k* clusters in which each observation is assigned to the cluster centroid to which it is closest. This approach was applied to the data 5,000 times with random starting centroids. ROIs that were unreliably clustered (frequency of primary cluster assignment greater than 2 standard deviations below the mean across all ROIs) were excluded from the final map. The number of clusters was identified by the Bayesian Information Criterion (BIC) [29], which balances the error of the fit with the complexity (i.e. number of parameters) of the model. We identified the appropriate cluster number as that number for which the change in the BIC with addition of another cluster was less than 5%.

## Results

**Figure 1:**
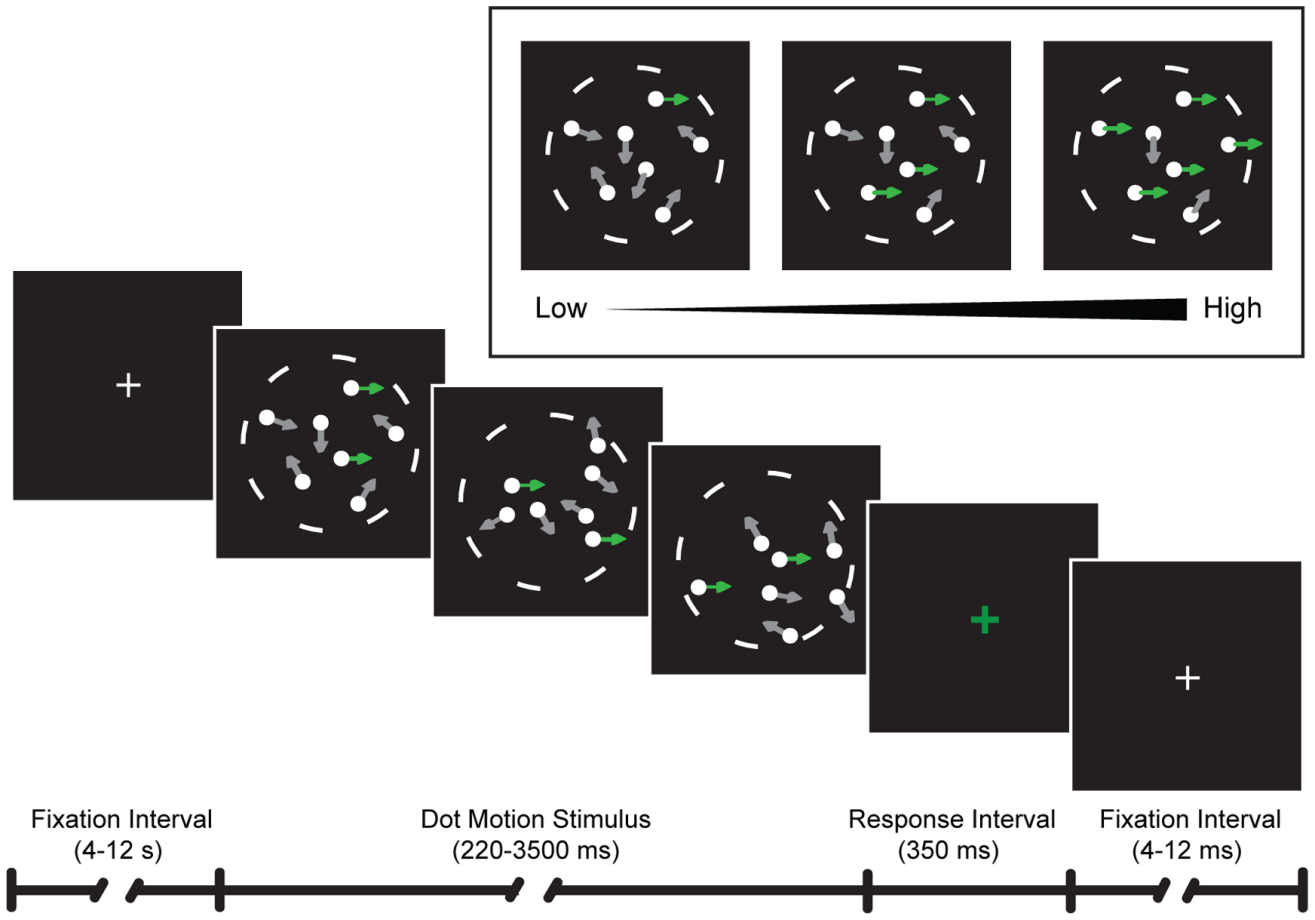
Task Design. Each trial consisted of a dot motion coherence stimulus displayed for 220 to 3500 milliseconds, with duration determined by the experimenter and defined by a gamma distribution equating the hazard rate across trials (see Methods). The motion coherence of the stimulus was also varied across trials (inset). To indicate that a response was required, the dot motion stimulus disappeared and a green fixation cross was displayed, after which subjects had 350 milliseconds to respond.

To better understand the neural mechanisms differentiating time on task from decision-related effects, we acquired fMRI data from five subjects performing a response signal task requiring discrimination of dot-motion direction. As described in Methods and in Figure 1, both duration of stimulus presentation and motion coherence were randomized across a range of values (duration: 220–3300 ms; motion coherence: 0%–100%). Duration values for each trial were selected from a distribution that equated the hazard rate, thereby rendering the duration of each trial less predictable [15]. Highly trained subjects were instructed to press one of two response buttons to indicate whether the motion was leftward or rightward. No performance feedback was provided during the scanning session. All subjects completed a minimum of 2592 trials.

### Behavioral Performance

**Figure 2:**
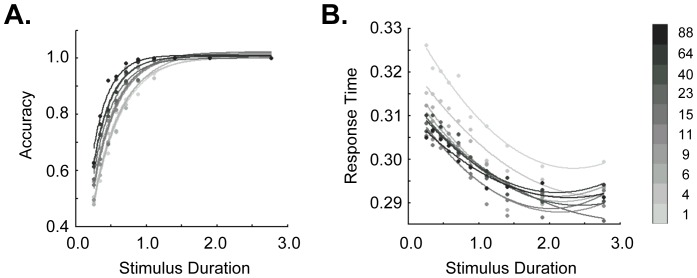
Behavior. All trials were divided by both motion coherence and duration into a total of 100 (10×10) bins. A. Accuracy increased with both increasing duration and increasing motion coherence. Curves represent the best-fitting exponential. B. Response time declined with both increasing duration and increasing motion coherence. Curves represent the best-fitting second-order polynomial.

Behavioral data for all subjects can be seen in Figure 2. In keeping with previous work in both humans and macaques, accuracy improved and response time declined as both duration and motion coherence increased. In particular, accuracy showed a strong main effect of both duration (F(9, 36) = 21.7, p<<10^−5^) and motion coherence (F(9, 36) = 156, p<<10^−5^). A significant interaction between duration and motion coherence (F(81, 324) = 2.09, p = 3.2×10^−6^) was driven in large part by a ceiling effect on accuracy at higher motion coherence values. Nonetheless, when the motion coherence bins encompassing 100% accuracy were removed, the interaction between them remained at trend significance (F(54, 216) = 1.37, p = 0.06). Across all motion coherences, linear regression demonstrated that these main effects were driven by a significant positive association between accuracy and both duration (r^2^ = 0.63, p = 0.006) and motion coherence (r^2^ = 0.77, p = 0.0008). For response time, significant effects were likewise seen for both duration (F(9, 36) = 5.1, p = 0.0002) and motion coherence (F(9, 36) = 14.9, p<<10^−5^). There was no interaction between these factors (p = 0.18). As indicated by linear regression, the main effect of motion coherence was accompanied by a strongly parametric effect of motion coherence on response time (r^2^ = 0.97, p<<10^−5^). In contrast, response time was not parametric with respect to duration (p = 0.19). Thus, subject performance showed the expected sensitivity to both factors.

### fMRI Analysis

**Figure 3:**
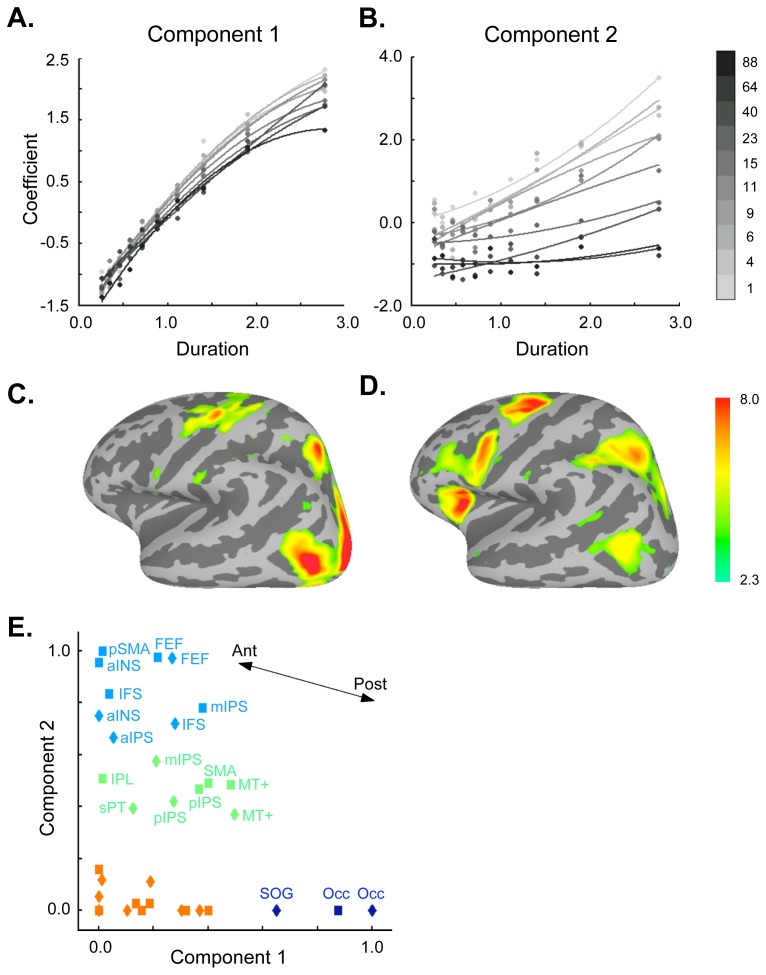
fMRI Results. A. Shown is the component that demonstrated the strongest effect of duration. Curves represent the best-fitting second-order polynomial. B. Shown here is the component that demonstrated both the strongest interaction between motion coherence and duration, and the strongest effect of motion coherence. C. The spatial map associated with component 1. D. The spatial map associated with component 2. The color bar represents Z scores and applies to both surfaces. E. Forty-one regions demonstrating a main effect of task were evaluated for their sensitivity to component 1 and component 2, each normalized to their respective maxima (Table 1). K-means clustering defined related regions within the component 1– component 2 space, where the value of each component for a given ROI was normalized to the maximum value of that component across all ROIs. Progressively more anterior regions showed less sensitivity to component 1, and more sensitivity to component 2, as indicated by the arrow. Labels are shown for ROIs demonstrating stronger component values (cyan, green, and navy clusters); the large number of regions that were minimally influenced by these factors (orange cluster) can be found, along with all labels and component values, in Table 1. Left-sided regions are represented by squares, right-sided regions by diamonds.

To identify areas sensitive to time on task, the perceptual decision, or their interaction, we applied independent components analysis to the beta values derived from binned duration and motion coherence parameters. Of the total of 11 independent components identified, the first task-related component showed the greatest parametric effect of trial duration (F(9,36) = 3422, p<<10^−5^; component 1, Figure 3A), and the second task-related component showed the greatest parametric effect of motion coherence (F(9,36) = 105, p<<10^−5^), as well as the largest interaction between duration and coherence (F(81,324) = 14.7, p<<10^−5^; component 2, Figure 3B). (Spatial distributions for the remaining nine independent components can be found in Figure S1.) The spatial distributions of these two components were also distinct. As evident in Figures 3C and 3D, component 1 showed activations that were greater in posterior brain regions and decreased in more anterior brain regions, while component 2 showed the reverse pattern.

To further investigate these differences, we plotted the relative magnitudes of each of these components within a two-dimensional space for each of 41 regions of interest derived from the main effect of task. K-means clustering was applied to these plots to define regions with similar sensitivities to either duration (component 1) or motion coherence (component 2; Figure 3E). This analysis identified an occipital cluster that was strongly sensitive to component 1 (dark blue); a cluster of primarily anterior regions including the anterior insula, preSMA, IFS, and the left mIPS, whose members were strongly driven by component 2 (light blue); a group of regions including the right mIPS whose sensitivity to both factors was intermediate (green); and a fourth cluster that, despite being active during the task, was relatively insensitive to either component (orange). In keeping with this result, we were able to define a posterior-to-anterior gradient of decreasing sensitivity to trial duration and increasing differential sensitivity to motion coherence using the MNI-derived y-coordinate for each region (Kendall’s τ = 0.45, p = 6.7×10^−5^; Figure 3E, arrow). Although both components contributed to this posterior-to-anterior gradient (as evidenced by the oblique angle of the vector in Figure 3), the effect was more sensitive to trial duration: duration was weighted 3.7-fold relative to motion coherence sensitivity. Thus, for regions in the anterior cluster especially, decision-related activity could not be well-explained by an effect of trial duration.

**Figure 4:**
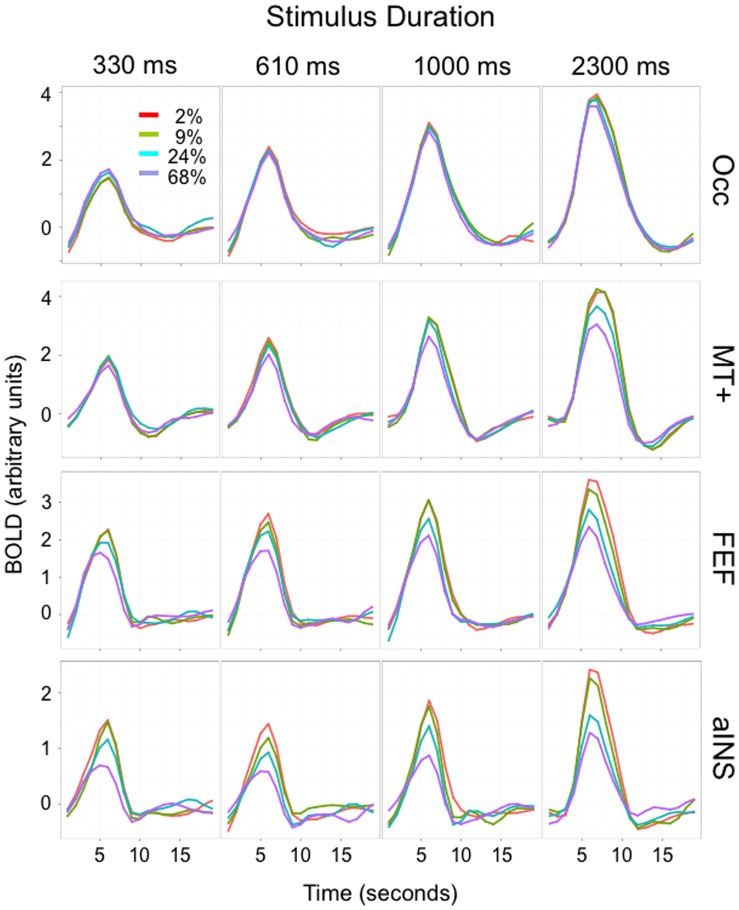
BOLD Time Courses. Shown here are time courses for four representative left hemisphere regions that together span the component space in figure 3E from posterior to anterior: Occ = occipital pole, MT+ = middle temporal region, FEF = frontal eye fields, and aINS = anterior insula. Trials are divided into 4 duration bins (at top: 0.33s, 0.61s, 1.0s, and 2.3s) and 4 motion coherence bins (inset: 2%, 9%, 24%, and 68%).

This gradient of sensitivity to the duration and motion coherence components could also be seen in group-averaged time courses (Figure 4). Although these time courses did not themselves distinguish the contributions of the two components, their relative influence in four representative left hemisphere regions spanning the component space from posterior to anterior (Figure 3E) was evident in the change in peak amplitude with trial duration, and the relative differentiation of the motion coherence values. Specifically, more posterior regions (Occ, MT+) demonstrated a stronger influence of the duration component, as reflected in peak amplitudes that increased progressively with trial duration, and a weaker influence of the motion coherence component, as reflected in reduced differentiation of motion coherence. In contrast, more anterior regions (FEF, aINS) demonstrated a stronger influence of the motion coherence component, as reflected in greater differentiation of the response to different motion coherence values (especially at shorter trial durations), and a weaker influence of the duration component, as reflected in a lesser change in peak amplitude with increasing trial duration. These qualitative findings were broadly consistent with the significance of ANOVAs related to duration and motion coherence, respectively. The effects of duration on peak amplitudes shown in Figure 4 were generally but not exclusively stronger in posterior ROIs (Occ: F_duration_ (3,12) = 34.0, p = 3.8×10^−6^; MT+: F_duration_ (3,12) = 14.1, p = 0.0003) than in more anterior regions (FEF: F_duration_ (3,12) = 20.0, p = 0.00006; aINS: F_duration_ (3,12) = 9.2, p = 0.002). Conversely, the effects of motion coherence on peak amplitudes were weaker in these two posterior ROIs (Occ: F_coherence_ (3,12) = 1.0, p = 0.45 (ns); MT+: F_coherence_ (3,12) = 6.7, p = 0.007) than in the two anterior ROIs (FEF: F_coherence_ (3,12) = 35.4, p = 3.1×10^−6^; aINS: F_coherence_ (3,12) = 27.8, p = 0.00001). The interaction term was strongest for the two intermediate regions (Occ: F_coh*dur_ (9,36) = 2.4, p = 0.03; MT+: F_ coh*dur_ (9,36) = 5.0, p = 0.0002; FEF: F_ coh*dur_ (9,36) = 7.2, p = 6.6×10^−6^; aINS: F_ coh*dur_ (9,36) = 1.7, p = 0.12 (ns).).

More generally, if the F values for the main effect of duration were compared with those for the main effect of coherence in these time courses across all 41 regions of interest, we were able to replicate the ICA finding (Figure 3E) of a posterior-to-anterior gradient of decreasing sensitivity to trial duration and increasing differential sensitivity to motion coherence (Kendall’s τ = −0.29, p = 0.003), despite the coarser division into 4 rather than 10 duration and coherence bins (see Methods). Duration was again weighted more heavily than motion coherence (1.37 fold). However, comparing F values for the main effect of duration with those for the interaction between duration and motion coherence reached only trend significance (Kendall’s τ = −0.17, p = 0.06).

## Discussion

In this study we used the response signal paradigm to behaviorally dissociate the duration between stimulus onset and motor response from the perceptual discriminability of the direction of dot motion. Using ICA, we showed a corresponding neurophysiological dissociation of the effects of time on task from decision-related processes tied to the strength of the perceptual stimulus. In so doing, we demonstrated that a cluster of regions including the anterior insula, preSMA, premotor cortex, and mIPS strongly represents decision-related processing that is independent of a “time on task” factor. Moreover, an anterior-posterior gradient defines the relative sensitivity of a given brain area to decision-related processes (as indexed by motion coherence) and time on task, respectively.

An important aspect of the response-signal paradigm used in this study is its ability to identify regions based on the extent to which their activation varied with each of the two task-related components. Because the consistent hazard rate renders the timing of the response signal unpredictable [15], the optimal strategy is to maintain attention throughout the duration of the stimulus. This behavioral requirement likely led to the strong time on task component we identified using ICA. By combining this aspect of the task with the requirements of the dot motion coherence decision, we were able to identify two independent components in the BOLD data strongly reflecting duration and motion coherence. Moreover, these components, which were selected based on their sensitivity to duration, or to motion coherence and the interaction between motion coherence and duration, respectively, were the two most strongly represented task components to arise from the ICA analysis.

Importantly, while the ICA analysis constrained the components derived from the beta values to be independent, it did not simultaneously constrain their spatial distributions to be independent and non-overlapping. This characteristic was particularly important when we considered the role of regions previously implicated in decision-related processes such as evidence accumulation (e.g. the middle IPS). While this region showed significant activity related to time on task, irrespective of motion coherence, it also demonstrated processing closely tied to the motion coherence of the stimulus. Moreover, along the posterior – anterior axis, mIPS was more strongly decision-related than MT+, which provides inputs to mIPS [30], and it was the most posterior region found in the anterior-most cluster of strongly decision-related areas. Of interest, right and left mIPS segregated into different clusters when only four clusters were present, suggesting that their functions might not be strictly homologous. Regardless, this conjunction of findings suggests that mIPS would be well-positioned to transform sensory inputs into decision-related representations [31].

Our analyses also demonstrated that more posterior regions including MT+ and posterior IPS are sensitive to both factors – i.e. perceptual discriminability and “time on task”. However, the ratio of the size of the motion component to the duration component in these regions was smaller than in mIPS. This finding suggests that the relative position of brain regions within the space defined by the duration and motion coherence components correlates with the probability of finding neurons that participate in processes such as evidence accumulation – specifically, that this probability is greater when greater motion coherence-related activity is distinguished from sensitivity to time on task. Future studies in macaque might profitably explore a range of such regions, as multiple regions are likely to encode the evidence for a perceptual decision [4]. Of particular interest would be to determine the relative percentages of cells that show selectivity for evidence accumulation in each of these areas, as these analyses suggest that the percentage of task-responsive neurons that do so might decrease as both the most posterior areas (which are most sensitive to time on task) and the most proximate motor areas (which are furthest removed from sensory representations) are interrogated.

These data also provide evidence that the activity of more anterior regions during the motion coherence decision cannot be easily explained by time on task arguments linked to stimulus duration. It has been argued, for example, that when subjects perform a Stroop task, BOLD activity in the dorsal anterior cingulate reflects time on task, as indexed by reaction times, rather than response conflict or some other process [12]. Here we provide evidence that this important consideration does not generalize to time on task as indexed by stimulus duration. In the anterior insula, for example, the component reflecting time on task is weakly expressed. One would thus have to argue that the time on task representation is limited to specific regions and specific tasks – itself an argument against a general time on task explanation – or, perhaps more parsimoniously, that activity in more anterior brain regions is more likely to be decision-related, in that it is bound to reaction time and not simply to stimulus duration.

These possibilities also confirm the more general importance of distinguishing time on task (or duty cycle) arguments from the duration of decision-making processes in other task paradigms. As we have argued previously [8], time can be a fundamental measure of the evolution of the decision, meaning that accumulator regions might be expected to show some effect of response duration. Areas such as the mIPS might show an effect interpreted as time on task, for example, as multiple noisy accumulators reach threshold at different times, leading to a generally progressive increase in BOLD. However, the strong presence of the other ICA component in the mIPS indicates that duration effects are also reflected in an interaction with motion coherence, as expected of a decision-related region. These ideas have at least two consequences for other task paradigms. If time on task and decision components remain undissociated, the presence of stimulus duration effects cannot be used to argue that a region fails to participate in decision-related processing. On the other hand, using reaction time as a covariate of no interest in GLM analyses may diminish the contributions of brain areas for which reaction time indexes important decision-related processes. These data do suggest that these effects will be less notable in more rostral areas, which show almost no effect of time on task in the current study. Consistent with the above ideas, the findings in this study accord well with other studies that have included duration considerations. Activity in the anterior insula in this study, for example, correlates well with activity that defines “decision commitment regions” in Ploran et al. [32] – but see also [7] – while other areas that show stronger effects of duration (PM, IFS, mIPS) correspond more strongly to their accumulator regions.

In the larger sense, these data also provide quantitative evidence for the commonly held idea that perceptual decisions identify a large-scale anterior-posterior gradient within the brain. Specifically, regions defined simply by their activity in the main effect of task segregate by anatomical location in the ICA analysis: effects of time on task are greater for more posterior/sensory regions, while effects of motion coherence are greater for more anterior regions. The strength of this gradient may depend in part on arbitrary factors – e.g. that the primary sensory regions in a visual task are quite posterior. It is possible, for example, that a task relying on somatosensory inputs, and therefore on a more anterior primary sensory area than the occipital cortex [33], may show a diminished gradient, while one that more strongly engages executive functions [34] may show an enhanced effect. As models of perceptual decision making implicitly demonstrate such a gradient [35], however, this analysis shows that such a gradient has a quantifiable basis, and confirms, along with supportive data from lesion studies in other paradigms [21], [22], for example, that it has validity in organizing the neurophysiological and cognitive bases for decision making in humans.

## Supporting Information

Figure S1 **Shown are seven slices in radiological convention (left = right) for each of the 11 independent components generated by the ICA analysis.** At top are the two components demonstrating the strongest correlation with task parameters as evaluated in the body of the paper: the component linked to task duration (D), and the component linked to both motion coherence and its interaction with duration (M). Below are shown the remaining 9 ICA components. In keeping with other applications of ICA, some of these components represent additional networks (e.g. component 2, which overlaps with areas in the default mode network that typically deactivate during task performance), while others appear to represent noise (e.g. component 4, which approximates the location of the ventricular system).
(TIF)
