# Supplementary figures and images for: Decomposing Effects of Time on Task Reveals an Anteroposterior Gradient of Perceptual Decision Regions
Source: PLoS One. 2013 Aug 19;8(8):e72074. doi: 10.1371/journal.pone.0072074 (PMC3747156; doi:10.1371/journal.pone.0072074)

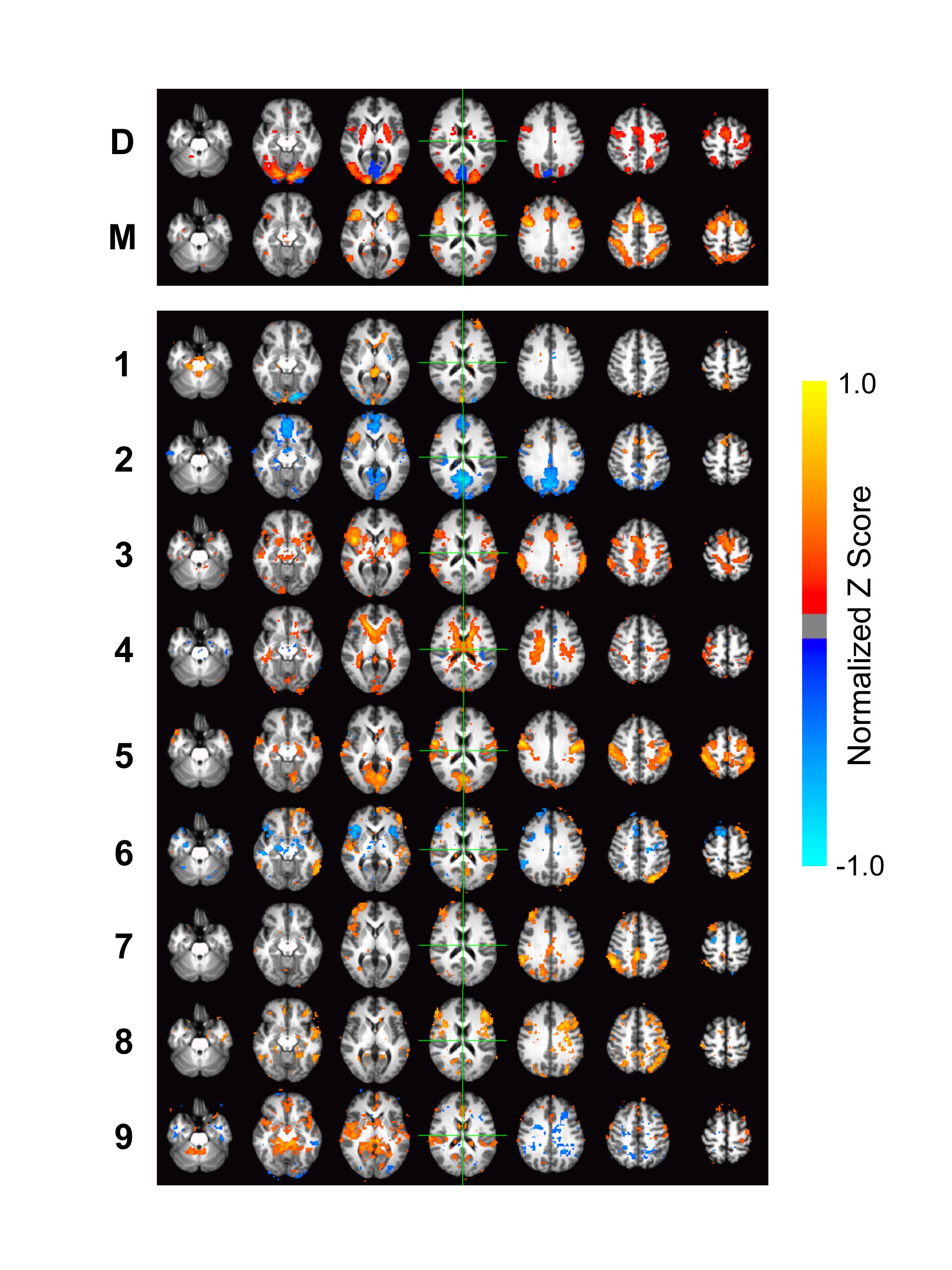

Supplement: Figure S1 — Shown are seven slices in radiological convention (left = right) for each of the 11 independent components generated by the ICA analysis. At top are the two components demonstrating the strongest correlation with task parameters as evaluated in the body of the paper: the component linked to task duration (D), and the component linked to both motion coherence and its interaction with duration (M). Below are shown the remaining 9 ICA components. In keeping with other applications of ICA, some of these components represent additional networks (e.g. component 2, which overlaps with areas in the default mode network that typically deactivate during task performance), while others appear to represent noise (e.g. component 4, which approximates the location of the ventricular system). (TIF) [file pone.0072074.s001.tif]
